# Solvent-Induced Assembly of One-Patch Silica Nanoparticles into Robust Clusters, Wormlike Chains and Bilayers

**DOI:** 10.3390/nano12010100

**Published:** 2021-12-29

**Authors:** Bin Liu, Serge Ravaine, Etienne Duguet

**Affiliations:** 1 Univ. Bordeaux, CNRS, Bordeaux INP, ICMCB, UMR 5026, 33600 Pessac, France; bin.liu-bin@u-bordeaux.fr; 2 Univ. Bordeaux, CNRS, CRPP, UMR 5031, 33600 Pessac, France; serge.ravaine@crpp.cnrs.fr

**Keywords:** silica, polystyrene, patchy particles, Janus particles, solvent-induced self-assembly, patch-to-particle size ratio, wormlike colloidal chain, colloidal bilayer, electron microscopy

## Abstract

We report the synthesis and solvent-induced assembly of one-patch silica nanoparticles in the size range of 100–150 nm. They consisted, as a first approximation, of silica half-spheres of which the truncated face was itself concave and carried in its center a polymeric patch made of grafted polystyrene chains. The multistage synthesis led to 98% pure batches and allowed a fine control of the patch-to-particle size ratio from 0.69 to 1.54. The self-assembly was performed in equivolume mixtures of tetrahydrofuran and ethanol, making the polymeric patches sticky and ready to coalesce together. The assembly kinetics was monitored by collecting samples over time and analyzing statistically their TEM images. Small clusters, such as dimers, trimers, and tetramers, were formed initially and then evolved in part into micelles. Accordingly to previous simulation studies, more or less branched wormlike chains and planar bilayers were observed in the long term, when the patch-to-particle size ratio was high enough. We focused also on the experimental conditions that could allow preparing small clusters in a good morphology yield.

## 1. Introduction

In recent decades, advances in both synthesis and characterization methods have made it possible to produce solid colloids that have become increasingly complex in terms of their composition, shape, and ability to interact with each other. In particular, the anisotropy of shape and/or chemical composition is particularly sought after programming the assembly modes. Then, patchy particles have attracted significant attention as potential building blocks often by simulation and sometimes through experiments [1,2,3]. In these situations, the patches, i.e., chemical or topological surface discontinuities, are attractive, while the rest of the colloid surface is neutral or repulsive.

Among these patchy particles, one-patch particles, also known as Janus particles for about three decades [4], are now easily achievable by numerous synthesis routes [5,6,7,8,9]. However, the study of their self-assembly only started about 15 years ago, both experimentally and by simulation starting with spherical particles, of which the Janus balance is geometrically quantified by the fraction of the surface occupied by the attractive patch (X).

The pioneering work of Granick and colleagues concerned the assembly of Janus spheres (X = 0.5) with an opposite electric charge into clusters, with the aggregation number (N) as high as 13, and their shape was corroborated by combined epifluorescence microscopy and Monte Carlo computer simulations [10]. The study was extended to Janus spheres with both a charged and a hydrophobic hemisphere that assemble in water first into small compact clusters (4 ≤ N ≤ 13) that link up into branched wormlike strings, as the increasing salt concentration enhances electrostatic screening [11]. Then, Miller and Cacciuto showed, only by simulation, that by increasing the hydrophobic patch area, particles self-assemble into small micellar clusters, wormlike structures, planar bilayers, faceted and fluid cages, and finally fcc crystals [12]. They stressed the importance of taking into account the dynamics of structure formation in the design process. Granick and colleagues reported that negatively charged spheres with one hydrophobic patch (X = 0.5) repeal one another electrostatically in pure water and aggregate irreversibly in salty water [13]. At low salt concentrations, they form small clusters (N ≤ 7) that polymerize into triple helices at higher salt concentrations and higher particle concentrations. Sciortino and colleagues simulated the influence of the reduction of X, they observed bilayers (X = 0.4), long wires (X = 0.3), and small micelles and dimers for lower X values [14,15], and they studied numerically the cooperative polymerization of the small clusters into the long wires [16]. They also showed that planar bilayers are not achievable for X = 0.5, because the particles cannot align side-by-side with all patches interacting sideways [17]. By way of compensation, they assemble into wrinkled bilayer sheets that are only observed if the square-well Kern–Frenkel attraction potential is maintained at a length of at least 20% of the particle diameter [18]. For larger patches (X = 0.6), they observed stable lamellar crystals made of stacked planar bilayers. More recently, Yi, Pine, and colleagues reported the fabrication of micron-sized bicompartment spheres made of polystyrene (PS) and poly[3-(trimethoxysilyl)propyl methacrylate] (PTPM) with a fine control of the Janus balance [19]. They successfully coated the PS counterpart with self-complementary DNA strands and studied experimentally and numerically the dynamic formation of assemblies as a function of X. All the structures formed are equilibrium structures, as they were thermally annealed in a temperature domain where the DNA interaction is dynamic and assemblies can reconfigure. They found small clusters (X = 0.3), trimer chains (X = 0.5), and bilayers (X = 0.6). For X = 0.3–0.4, the clusters serve to nucleate and grow dimer chains. Moreover, by introducing also different self-complementary DNA strands with different melting profiles on the other hemisphere and a toehold displacement strand in a solution, they switched the Janus balance in response to the temperature and realized reconfigurable, thermally reversible transitions between chains and bilayers [20].

Dumbbell-shaped particles consist of two more or less interpenetrated spherical particles. When one of them is attractive (A), while the other is non-attractive (NA) or even repulsive, they can be also considered as Janus particles characterized by the patch-to-particle size ratio S = D_A_/D_NA_, and sphere separation L = 2d/(D_A_ + D_NA_) with d as the distance between the two sphere centers. Whitelam and Bon studied numerically the self-assembly in water of amphiphilic dumbbells and observed bilayers for S of ~1 and micelles for lower S values, becoming more spherical when S tends to 0.5 [21]. The rate of interpenetration of the patch into the particle L seems to be a less critical parameter. More recently, by applying the interaction range of half the particle diameter, Avvisati, Dijkstra, and colleagues studied the influences of the volume fraction ranging from 0 to 0.25, S, and L [22]. For S = 0.97, they obtained spherical micelles for L < 0.1, elongated micelles for 0.1 < L < 0.2, vesicles for L = 0.2 and bilayers for L > 0.2, consistently with the fact that the lower the sphere separation, the more directional the attraction. For S = 0.8, upon increasing L, the structures change from spherical micelles to elongated micelles and ultimately, at sufficiently high volume fractions, to bilayers. Lastly, for S = 1.05, they found bilayer formation on a wide range of L values and volume fractions. Still by simulation, Paul and Vashisth observed the formation of bilayers, elongated micelles, and spherical micelles, when S values are 1, 0.7, and 0.55, respectively [23]. From an experimental viewpoint, Kang and Honciuk prepared several batches of dumbbells made of PTPM spheres with hydrophobic poly(t-butyl acrylate) patches with different S and L values. By assembling in 90/10 (*v*/*v*) water/ethanol mixtures under shear, they observed monolayer capsules of which the radius of curvature is greater as S is greater [24]. Free-standing bilayers are also obtained when S is ~1. They reported also the formation of small clusters, elongated micelles, elongated vesicles, and giant vesicles with N up to 8000 [25]. Although the resulting structures appear strong enough to be observed, the authors mentioned the deformability of the polymeric patches but did not specify or show whether the patches partially merged when in contact under these experimental conditions.

Obtaining assembled structures that can be preserved, manipulated and used is yet just as important as understanding the rules of their design and the dynamics of their formation. To fabricate more robust assemblies, while preserving the assembly reversibility that is mandatory for repairing stacking faults as the structures are growing, several options were investigated. In particular, it was envisioned that the attractive patch should be also sticky, capable of merging with similar ones and leading to robust bonds when the structure is frozen, e.g., due to the drop in temperature or the quality of the solvent. This was experimentally investigated with hard–soft dumbbells by some groups. For instance, Bon and colleagues observed well-defined clusters (2 ≤ N ≤ 5) formed in water from micron-sized asymmetric dumbbells (S = 0.6–0.8) made of PS and poly(n-butylacrylate) patch when the poly(vinylpyrrolidone) stabilizer desorbs from the particles, making the soft patches capable to merge upon contact through collision [26]. We also reported the self-assembly of silica/PS dumbbells (in the size range of 100–200 nm) when they are incubated into mixtures of good/bad solvents for PS, such as dimethylformamide/ethanol mixtures [27]. We studied the influence of S values from 0.32 to 1, the good solvent fraction, and other experimental parameters. We obtained not only highly regular clusters (2 ≤ N ≤ 6), of which the morphology was governed by the patch deformation and coalescence, energetically driven by surface energy minimization, but also micelles probably entrapping some dumbbells in their cores.

**Figure 1:**
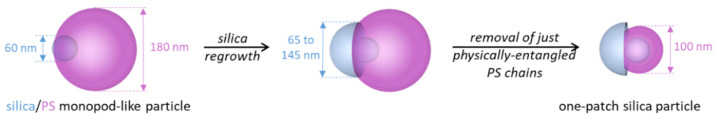
Two-step synthesis pathway to obtain silica particles with one PS patch of a tunable relative size from silica/PS monopod-like nanoparticles.

We report here another study using differently shaped one-patch silica/PS nanoparticles (Figure 1) that allowed us to fabricate, for the first time at this scale and as robust entities, extended assemblies predicted by simulation that are chains and bilayers. Because their patch consisted essentially of grafted PS chains, the silica particles were initially dispersed in a good solvent for PS, i.e., tetrahydrofuran, and its quality was lowered by adding ethanol as a PS non-solvent. As at this scale the observation was only possible by electron microscopy, we took numerous samples over time and statistically analyzed the images to determine the kinetics of the assembly mechanism and thus the “reactivity” of the one-patch particles as a function of their patch-to-particle size ratio S. Lastly, we determined the optimal experimental conditions to maximize the formation of specific clusters, e.g., dimers, trimers, or tetramers, made of similar silica nanoparticles or silica nanoparticles with different S values.

## 2. Materials and Methods

### 2.1. Materials

Tetraethoxysilane (TEOS; ≥99%), styrene (≥99%, with ca. 50 ppm 4-tert-butylcatechol as a stabilizer), sodium persulfate (Na_2_S_2_O_8_; ≥99%), polyethylene glycol nonylphenyl ether (Synperonic^®^ NP30), sodium dodecylsulfate (SDS; 99%), and tetrahydrofuran (THF; ≥99% with 250 ppm butylhydroxytoluene as an inhibitor) were purchased from Sigma-Aldrich (Saint-Quentin Fallavier, France). Methacryloxymethyltrimethoxysilane (MMS; 95%) was purchased from ABCR (Karlsruhe, Germany). Ammonium hydroxide (NH_4_OH; 28–30% in water) and ethanol (99%) were provided by Atlantic Labo (Bruges, France). Deionized water with a resistivity of 18.2 MΩ·cm at 25 °C was obtained from a Milli-Q system (Merck Millipore, Darmstadt, Germany). All chemicals were used without further purification.

### 2.2. Synthesis of One-Patch Silica Nanoparticles

One-patch silica nanoparticles were derived from silica/PS monopod-like nanoparticles after silica regrowth and removal by the dissolution of the physically entangled PS macromolecules (Figure 1).

Silica nanoparticles with an average diameter of 60 nm and a polydispersity index of 1.02 were obtained by TEOS hydrolysis/polycondensation according to a two-stage protocol already published [28]. At the end of the synthesis, the silica surface was functionalized with methacryloxymethyl functions by reacting with MMS at room temperature for 3 h and then one more hour at 90 °C under stirring. The added MMS amount was calculated for tuning the nominal grafting surface density at 1 function/nm^2^. The mass concentration of the silica particles was checked by the dry extract method.

Then, the MMS-modified silica nanoparticles were used as seeds for the seed-growth emulsion polymerization of styrene to obtain silica/PS monopods with a PS pod diameter of about 180 nm, according to a protocol adapted from those already described in our previous publications [29,30]. Typically, in a 100 mL three-neck round flask equipped with a condenser, 50 mL of emulsion were prepared by mixing Synperonic^®^ NP30 (2.97 g/L) and SDS (0.03 g/L), the MMS-modified silica nanoparticles (1.8 × 10^16^ part/L), and styrene (100 g/L) in water. The emulsion was thoroughly deaerated by bubbling nitrogen under stirring. The temperature was increased to 70 °C, and then the styrene polymerization was triggered by adding 1.3 mL Na_2_S_2_O_8_ solution (0.1 g dissolved in 4 mL water). After 6 h of polymerization, the sample was transferred into a 50 mL tube and stored at 4 °C.

The regrowth of the silica core was performed as follows: Ethanol (9.1 mL), ammonia (0.7 mL), and the monopod dispersion (0.2 mL, 1.8 × 10^16^ part/L) were introduced into a 25 mL flask, and the mixture was homogenized by stirring for 5 min. Then, 70, 100, and 200 μL TEOS were added, and the reaction was left under stirring at room temperature for 15 min. The particles were purified by three cycles of centrifugation/redispersion in ethanol (12,000× *g*; 8 min, 15 mL). The TEM analysis showed that, in such conditions, the silica diameters of the monopods for 70, 100, and 200 μL TEOS were enlarged to 65, 85, and 115 nm, respectively. To obtain a higher diameter of 145 nm and avoid the occurrence of the secondary nucleation of silica, the addition of 500 µL TEOS was sequenced in three doses of 200, 100, and then 100 μL, including after each addition/reaction stage a purification step by centrifugation/redispersion as previously described.

Lastly, the removal of the PS macromolecules that were just physically entangled to reduce the PS pod diameter from 180 to 100 nm was performed for each batch by three centrifugation/redispersion cycles in THF (12,000× *g*; 10 min; 20 mL). The final concentration of the one-patch silica nanoparticles in THF was adjusted to 3.6 × 10^14^ part/L.

### 2.3. Solvent-Induced Colloidal Self-Assembly

The assembly of the silica nanoparticles was triggered by making sticky the PS patch by adding a non-solvent to the particles initially dispersed in a good solvent for PS, according to a strategy already investigated by us with silica/PS dumbbells displaying other morphologies [27]. The experiments were routinely carried out in 1.5 mL glass bottles closed with a polypropylene cap and gently stirred on a roller mixer at 60 rpm and at room temperature all along the incubation. For reducing the quality of the THF solvent, a certain volume of ethanol was added to reach a fraction of 50 vol% and a total volume of 500 µL, except for some experiments specified in the text. The assembly kinetics was monitored by regularly collecting 30 µL samples from the bottle and direct deposition on TEM grids for liquid evaporation before observation. A study, not reported here, showed that freezing the assemblies during the evaporation process by the dilution of the collected sample in ethanol was useless. On the other hand, for the long-term preservation of the assemblies, freezing their structures was mandatory and successfully performed by pouring a full-incubation medium into 20 mL cold ethanol and immediate storage at 4 °C.

### 2.4. Characterization by TEM

TEM images were obtained on a Hitachi H600 or JEOL 1400 microscope operating at 75 and 120 kV, respectively. Typically, the particles collected in the course of the assembly were dropped directly on carbon-coated copper films (300 mesh), and the liquids were allowed to evaporate at room temperature in a fume hood. Statistics from image analysis were performed using at least 500 nano-objects for the seeds, 200 for the one-patch silica nanoparticles, and 200 for their assemblies.

## 3. Results and Discussion

### 3.1. Synthesis of One-Patch Silica Particles with the Patch-to-Particle Size Ratios from 0.69 to 1.54

Thanks to our know-how developed over the last 10 years, we prepared a batch of silica/PS monopods by the emulsion polymerization of styrene in the presence of 60 nm silica seeds with a morphology yield of 98% (Figure S1) [29,30]. To promote the formation of monopods, the wettability of the growing PS nodule on the silica seed was enhanced thanks to an optimized surface grafting density of the methacryloxymethyl groups of 1 function/nm^−2^. The by-products (2%) were also monopods with not one, but two silica seeds attached to each other. Because of this very low prevalence and for simplification reasons, they were excluded from forthcoming statistics, i.e., the batches of one-patch particles were considered pure.

Four batches of one-patch silica particles were indeed obtained from this batch of silica/PS monopods after a two-step pathway. The first step was the regrowth of the silica from the surface of the initial silica seed that protruded out of the PS pod. The diameters of the obtained silica protrusion, i.e., 65, 85, and 115 nm, were tuned by the added amount of TEOS. To obtain a higher diameter of 145 nm and avoid the occurrence of the secondary nucleation of silica, the addition of TEOS was sequenced in three stages. The second step was the removal of the only physically entangled PS chains by dissolution in THF to thin the PS pod to 100 nm around the initial silica seed. Thus, this irremovable PS layer was essentially made up of covalently grafted PS macromolecules, resulting from the copolymerization of styrene with the methacryloxymethyl groups initially grafted onto the silica seeds.

**Figure 2:**
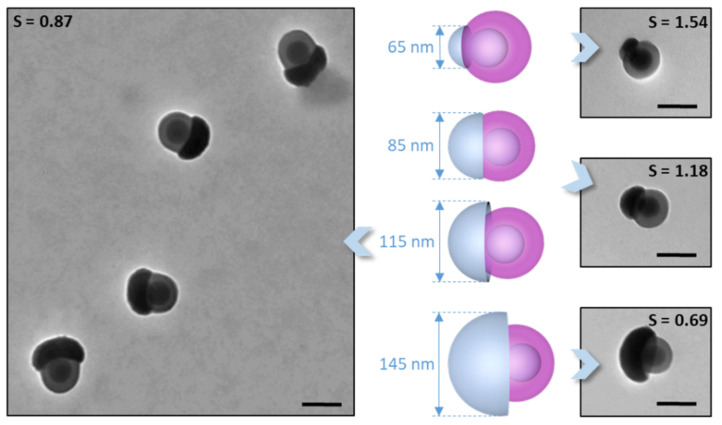
Representative TEM image of silica nanoparticles of sizes from 65 to 145 nm with a single PS patch of which the diameter was 100 nm. As a consequence, the patch-to-particle size ratio S values of these particles varied from 1.54 to 0.69. Scale bars: 100 nm. Due to their higher electron density, silica particles had a significantly more contrast and therefore appeared darker than PS ones of a similar size. A low-magnification image of the one-patch silica nanoparticles with S = 0.69 is displayed in Figure S2.

Figure 2 shows the typical TEM images of the as-obtained one-patch particles, of which the patch-to-particle size ratio S values were 1.54, 1.18, 0.87, and 0.69, respectively. We can see that, as anticipated in Figure 1, the shape of the silica particle was more of a half-sphere with the PS patch emerging from the truncated part. The statistical analysis of these images showed that these batches of one-patch silica nanoparticles were morphologically as pure as those of the starting batch of monopods, i.e., about 98%. Moreover, as this series of one-patch particles was obtained from the same batch of silica/PS monopods, we assumed that their PS patches were identical in terms of the average macromolecule length and quantity. The latter was confirmed by the consistency of the thickness of the PS shells, i.e., about 20 nm, observed in the TEM images. Thus, we considered hereafter that the patches had the same stickiness, all else being equal, as the solvent/non-solvent mixture composition, temperature, and particle concentration.

### 3.2. Self-Assembly Behavior of One-Patch Silica Particles with a Patch-to-Particle Size Ratio of 0.87

Because the PS chains were grafted to the surface of the initial silica seed, they behaved differently, depending on the nature of the liquid in which the particles were dispersed. In a PS non-solvent, such as ethanol or water, the chains are collapsed around the silica to minimize interactions with the liquid by maximizing interactions between the monomer units. This collapsed state of the polymer shell is similar to that observed in the TEM images taken in a vacuum in Figure 2. On the contrary, in a good PS solvent, such as THF or dimethylformamide, the monomer unit/solvent interactions replace the interactions between monomer units, which leads the polymer chains to extend as far as possible from the silica surface, which they cannot leave because they are chemically grafted there. The polymer shell thus takes the form of a polymer corona swollen with the solvent, which results in a strong increase in volume. Thus, when two particles come close enough, their polymer coronas can transiently interpenetrate. By lowering the quality of the solvent, e.g., by adding a miscible non-solvent, the attraction between the macromolecules can be promoted again: the swelling rate decreases, and two coronas in close proximity attract each other. Thus, the sticky character of the patch is explained on the one hand by the presence of the good solvent, which ensures a minimal mobility of the macromolecules to facilitate the interpenetration of the two coronas, and on the other hand by the degree of attraction between the macromolecules, promoting the attraction, making the interpenetration less reversible, and thus increasing the life span of the assembly. This one can become permanent, if the good solvent is eliminated and if the temperature is maintained below the temperature of the glass transition (case of amorphous polymers) or of the melting temperature (case of semi-crystalline polymers). With amorphous PS, the assemblies can be maintained at temperatures up to 100 °C. This type of macromolecular entanglement assembly is widely known in the context of block copolymers and leads to spectacular assemblies [31].

Our previous work on the silica/PS dumbbell assembly has shown that the optimal composition of a good solvent/non-solvent mixture depends on the system to be assembled, in particular the amount of polymer to be made sticky and the presence of non-grafted macromolecules which imposes to the protocol to disperse the patchy silica particles in the non-solvent and the good solvent to be added [27]. Generally speaking, the larger the size and amount of polymer patches, the larger the fraction of the good solvent must be.

**Figure 3:**
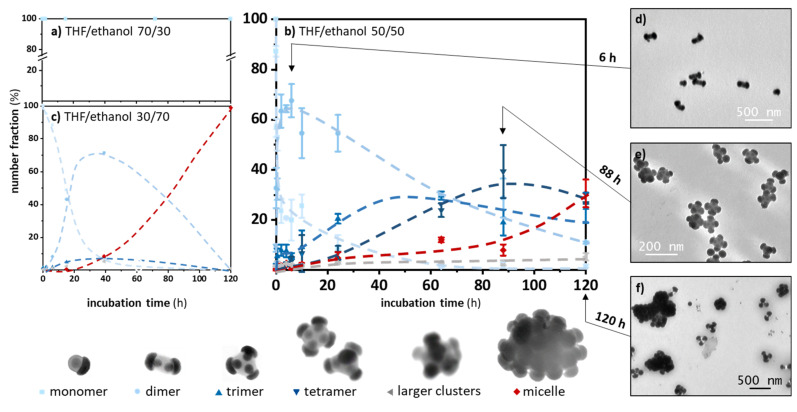
Evolution with the incubation time of the number distribution of silica monomers (S = 0.87), dimers, trimers, etc., as determined by the statistical analysis of the TEM images for different fractions of ethanol in the THF/ethanol mixture: (**a**) 30 vol%; (**b**) 50 vol%; and (**c**) 70 vol% (the dotted curves are only guides for the eyes). Experimental conditions: silica particle concentration, 1.8 × 10^14^ L^−1^; temperature, 20 °C. The representative TEM images of the silica particles present in the incubation medium after 6 h (**d**), 88 h (**e**), and 120 h (**f**) corresponding to some of the samples collected for building the graphs in (**b**).

In the present study with these new one-patch particles only made of grafted macromolecules, we showed that reaching 30 vol% ethanol was not sufficient for the patches to attract and/or for macromolecular interactions to be sufficient to maintain the assembly (Figure 3a). Indeed, no assembly was obtained after five days of incubation. On the other hand, when the ethanol fraction reached 50 vol% (Figure 3b) or 70 vol% (Figure 3c), the patchy particles (monomers) rapidly disappeared in favor of assemblies of two silica particles (dimers, N = 2), and the average number of aggregation progressively increased with the appearance of trimers, tetramers, and micelles, for which the number of constituent particles became difficult to determine from the TEM images. Comparing the graphs of Figure 3b,c, we can see that the proportions of the assemblies with intermediate N values, e.g., trimers and tetramers, were greater when the volume fraction ethanol was 50%, and that is why we used equivolume THF/ethanol mixtures for the rest of the study.

A more detailed analysis of the graphs in Figure 3b allowed a better understanding of the assembly mechanism, which was comparable to that of a step-growth polymerization: the monomers progressively disappeared for the benefit of more or less provisional assemblies such as dimers, then trimers, and tetramers, of which the maximum concentrations were reached after approximately 6, 50, and 90 h, respectively. After five days, the incubation medium consisted of dimers (~12%), trimers (~20%), tetramers (~30%), and micelles (~30%). The remainder consisted of a few monomers and clusters with N > 4.

As shown at the bottom of Figure 3, the polymeric phase that bound the different silica particles within the different clusters was particularly homogeneous, which proved that the coalescence by interpenetration of the macromolecules grafted to the different patches was real and effective. Moreover, these silica clusters were relatively symmetrical, even if the assembly of the silica particles by the coalescence of their PS patch did not produce perfect repulsion figures: straight for dimers and equilateral triangles for the trimers. Indeed, it happened that bent dimers, dimers where the silica particles do not face each other exactly, and triangles other than equilateral ones were observed. This showed that the steric hindrance of the silica particles or the repulsion forces between them were insufficient to perfect the geometry of the clusters. In the case of tetramers, tetrahedral shapes were observed, but mostly diamond-like shapes. This may be due to the orientation of the tetramers on the TEM grid, but also to a certain deformability of the clusters at the time of grid preparation due to the presence of residual THF molecules within the PS component and serving as a plasticizer, as previously reported by us for other silica/PS multipods [32]. Thus, the deviations from the symmetry observed in the clusters, regardless of the N values, could occur at the time of the grid preparation and not pre-exist in the solution. Only time-consuming studies by dynamic light scattering or cryo-electron tomography could confirm this hypothesis.

Finally, to verify that these clusters were metastable, we checked that the disassembly of the micelles into monomers was complete in a few minutes as soon as a large amount of THF was added to the dispersion (Figure S3).

### 3.3. Influence of the Patch-to-Particle Size Ratio on the Self-Assembly Capacity and Achieved Structures

**Figure 4:**
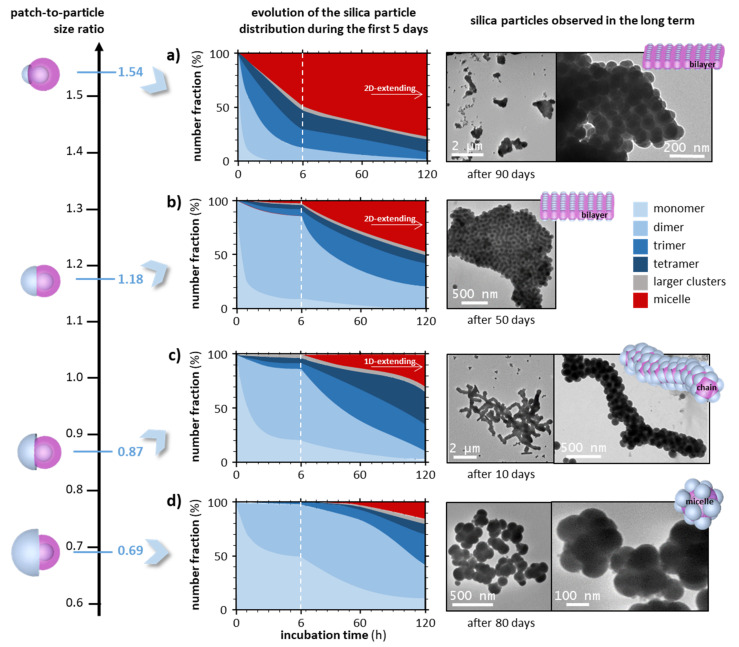
Evolution with the incubation time of the number distribution of silica monomers, dimers, trimers, etc. as determined by statistical analysis of the TEM images for different S values: (**a**) 1.54; (**b**) 1.18; (**c**) 0.87; and (**d**) 0.69. Experimental conditions: equivolume THF/ethanol mixture; silica particle concentration, 1.8 × 10^14^ L^−1^; temperature, 20 °C. The representative TEM images of the silica particles present in the incubation medium in the long term are shown. Some extra images captured by scanning electron microscopy are displayed in Figure S5.

To study the influence of the S parameter, we performed the same assembly experiments in equivolume THF/ethanol mixtures and identical concentrations from batches of one-patch silica particles with diameters of 65, 85, and 145 nm, corresponding to the S values of 1.54, 1.18, and 0.69 respectively. Using the same methodological approach of sampling over time and the statistical analysis of TEM images, we compared the rate of appearance and proportion of each type of cluster for incubation times up to 120 h (Figure 4).

The first observation was that the larger the relative size of the patch, i.e., the larger the patch-to-particle size ratio, the more quickly the monomers assembled and therefore disappeared in favor of clusters of higher N, which themselves evolved more rapidly. As we have assumed that their stickiness was identical (see Section 3.1), this difference in “reactivity” was essentially due to the steric hindrance of the silica particle: the smaller the silica particle, the more frequent the collisions between objects involving a patch against a patch and therefore the higher the probability of the assembly. It was also likely that the larger the silica particle, the lower its velocity and therefore the lower the frequency of collisions.

The second observation concerned the size of the assemblies obtained over long incubation periods. Whatever the S value, we observed the formation of spherical micelles, which did not evolve for S = 0.69. However, the higher the S value, the more these micelles evolved into larger objects. They elongated in the form of chains for S = 0.87 or extended into bilayers for S = 1.18 or 1.54. These extended structures, already predicted by previous simulation studies [12,14,15,16,17,21,22,23], have also been observed [11,13,19,20,24], but never for this size of particles and this type of interaction. As already discussed in the introduction, these extended structures require that the patch can interact not only with the patch of particles facing it, but also with sideways. Indeed, the smaller the relative size of the patch, the smaller the number of bonds it is able to establish with other similar particles and therefore the smaller and more spherical the clusters. On the contrary, the lower the steric hindrance of the silica particles, the more the assemblies have the possibility to grow. Although the geometry of our dumbbells is different from those studied previously and the interaction potentials applied in the simulations vary from one study to another, we roughly evidenced the same types of assembly for the same range of S or X values.

These extended assemblies were initially observed after incubation times of several tens of days. However, we showed with one-patch silica particles having an S value of 1.18 that similar bilayers might be obtained after only 90 min under ultrasounds (Figure S4).

In the chains and bilayers, we observed (Figure 4a–c) the silica particles were more or less regularly positioned. In particular, the chains with an approximate diameter of 300 nm showed many defects leading to branching, in contrast to the dimer chains and trimer chains observed with the DNA-assisted assemblies of microparticles [19].

### 3.4. Targeting Specific Assemblies with Low Aggregation Numbers

With the knowledge of these mechanisms, we tried to find the experimental conditions that would allow us to favor the formation of specific clusters, preferably those with a low N, and therefore to obtain them with the best possible morphology yield. We previously verified that it is possible to freeze the structure of assemblies by diluting 20 times in ethanol (Figure S6).

Favoring the formation of dimers is not very complicated, since they are the first clusters formed as soon as two monomers encounter the opportunity to assemble. However, the difficulty lies in finding the optimal incubation time, so that monomers with the maximum number react before too many dimers evolve into trimers, etc. According to Figure 4, it was for silica particles with S = 0.87 and 1.18 that the number fraction of dimers was the highest, i.e., 65% for 4–6 h and 75% for 2–6 h of incubation, respectively. The choice of the exact incubation time depends on the nature of the by-products tolerated, mainly monomers at the beginning of the period or trimers, tetramers, etc. at the end of the period.

**Figure 5:**
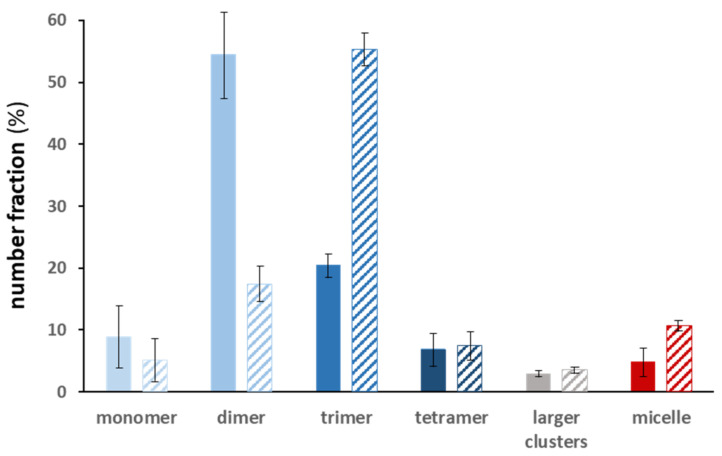
Number distributions of silica monomers, dimers, trimers, etc. as determined by the statistical analysis of the TEM images after 24 h of incubation of 115 nm one-patch silica particles (S = 0.87). The hatched histogram correspond to the same experiment including the addition of 50% more monomers after 8 h of incubation, with all else being equal: equivolume THF/ethanol mixture; silica particle concentration, 1.8 × 10^14^ L^−1^; temperature, 20 °C.

Still according to Figure 4, the conditions for obtaining batches enriched in trimers or tetramers were a priori less favorable than those for obtaining dimers. Interestingly, we nevertheless found a trick to artificially promote the formation of trimers, particularly with the 115 nm monomer batch (S = 0.87). This involved adding 50% more monomers after eight hours of incubation, with all else being equal, comparing the proportion of trimers obtained after 24 h (Figure 5). It was found that the fractions of dimers and trimers were reversed. This second quantity of monomers allowed the majority population of dimers to be transformed into trimers, thus achieving a record morphology purity of 55% for trimers.

Finally, it seemed relevant to study the possibility of preparing dissymmetric dimers or even trimers by incubating mixtures of monomers with silica diameters of 115 and 65 nm, corresponding to the S values of 0.87 and 1.54, respectively, with both concentrations being half the same as in the previous experiments. After two hours of incubation, we counted 7% monomers, 60% dimers, and 26% trimers, with the rest being the usual clusters of higher N (data not shown). The very small fractions of monomers (1%), homodimers (1%), and homotrimers (<1%) consisting exclusively of silica particles with S = 1.54 confirmed that the larger the patch-to-particle size ratio, the shorter the lifetime of the monomers, dimers, and trimers, which also explained why the larger clusters and micelles comprised mainly small silica particles. Conversely, silica particles with S = 0.87, known to be less “reactive”, constituted the majority of dimers (34% homodimers and 25% heterodimers) and trimers (3% homotrimers and 23% heterotrimers). In order to increase the proportion of heteroclusters, the higher “reactivity” of the smaller silica particles would have to be compensated either by lowering their concentration or by adding them several times, following the strategy described above to increase the amount of trimers.

**Figure 6:**
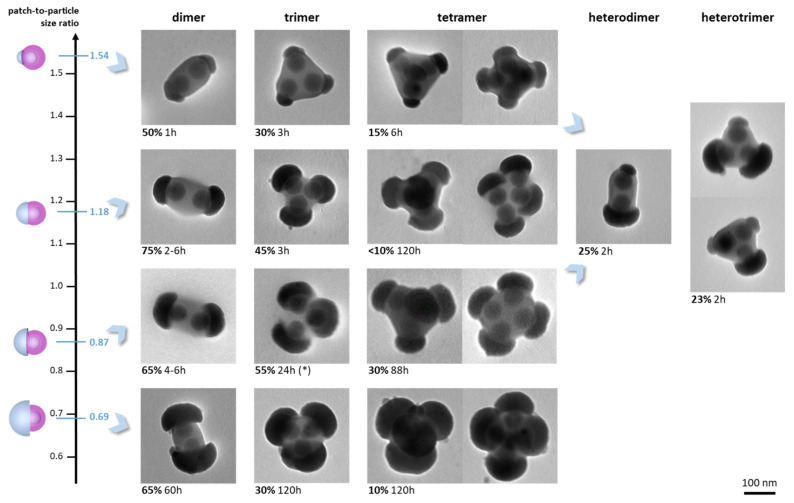
Representative TEM images of the dimers, trimers, and tetramers obtained from one-patch silica nanoparticles by the solvent-induced assembly. The optimum morphology yield and the incubation time after which it was observed (cf. Figure 4) are indicated, excepted for the trimers (*), which were optimized by the sequential addition of monomers (cf. Figure 5). Experimental conditions: equivolume THF/ethanol mixture; silica particle concentration, 1.8 × 10^14^ L^−1^; temperature, 20 °C.

Figure 6 summarizes the diversity of dimers, trimers, and tetramers we obtained in this study and the morphology purity achieved. These values could certainly be increased by implementing the trick of sequential monomer addition. Nevertheless, it seems compromised to obtain pure batches without considering extra steps of purification, for example by gradient density centrifugation. Figure 6 also clearly shows that the polymer phase between the silica particles had a uniform contrast and was therefore continuous and homogeneous. This is a clear confirmation of the interpenetration of the macromolecular coronas of the patches achieved thanks to the physical entanglement of the PS chains.

## 4. Conclusions

This study showed that the multistep synthesis of one-patch silica nanoparticles in the approximate form of a silica half-sphere carrying, in the middle of its truncated face, a patch made of grafted PS chains was efficient. The patch-to-particle size ratio can be easily adjusted by the regrowth degree of the silica. Their morphological purity reached 98% and depended essentially on that of the starting batch of monopod-like silica/PS particles.

The self-assembly of the one-patch silica nanoparticles was shown to be particularly successful for equivolume mixtures of THF and ethanol. The larger the patch-to-particle size ratio, the faster the assembly, i.e., the higher the probability that the collision between two particles was patch-to-patch. Thus, the smallest silica particles proved to be the most “reactive”, as well as the ones that allowed achieving the most extended assemblies, i.e., wormlike chains and especially bilayers, as expected by the previous simulation works. To the best of our knowledge, this is the first time that this type of colloids has been obtained from elementary blocks with sizes from 100 to 150 nm and in a mechanically robust form ensured by the interpenetration of the macromolecules of the different patches. However, the distribution of the silica half-spheres on the surface of these objects was not specifically organized. To make a good arrangement, it would probably be necessary to draw on the simulation work of Sun and colleagues, who numerically investigated the assembly of soft Janus particles capable to deform and overlap, e.g., micelles, microgels, and dendrimers [33]. They showed that, as the adhesion energy increases, the structures become more symmetrical: wormlike chains evolve into double and even single helices, and bilayers develop into tetragonal bilayers. From an experimental point of view, this could probably be achieved by an annealing step, either in temperature or in the presence of a small amount of a solvent or plasticizer, to restore mobility to the macromolecules while making silica particles electrostatically repulsive to each other.

Moreover, we have shown that this solvent-induced assembly pathway associated with this novel morphology of one-patch silica particles allows the preparation of colloids with a low aggregation number, e.g., dimers, trimers, and tetramers, and that it is to some extent possible to increase their morphological purity by delayed particle additions. However, obtaining perfectly pure batches will most likely require a purification step. Finally, the mixing of silica particles with different sizes has made it possible to obtain heterodimers and heterotrimers that are also asymmetric objects and could play a promising role in other assembly strategies.

Finally, this study carried out with the model case of silica/PS one-patch nanoparticles now deserves to be extended with more functional nano-objects capable of providing optical, magnetic, catalytic properties, etc.

## Data Availability

The data presented in this study are available from the corresponding author on request.

## Supplementary Materials

The following are available online at https://www.mdpi.com/article/10.3390/nano12010100/s1, Figure S1: TEM image of silica/PS monopod-like nanoparticles used as precursor colloids, Figure S2: Low-magnification TEM image of 145 nm silica particles with a single 100 nm PS patch, Figure S3: TEM images showing how micelles obtained from 115 nm silica particles were fully disassembled in 10 min after the addition of THF, Figure S4: TEM image of bilayers obtained from 85 nm silica particles after 90 min of incubation in the equivolume THF/ethanol mixture under ultrasounds, Figure S5: Scanning electron microscopy images of spherical micelles and wormlike chains obtained from 115 nm or 145 nm silica particles, Figure S6: TEM images showing that the structure of silica dimers may be frozen by dilution in ethanol and preserved up to 52 days at 4 °C.
