# Supplementary material for: Solvent-Induced Assembly of One-Patch Silica Nanoparticles into Robust Clusters, Wormlike Chains and Bilayers
Source: Nanomaterials (Basel). 2021 Dec 29;12(1):100. doi: 10.3390/nano12010100 (PMC8747025; doi:10.3390/nano12010100)
Supplement: Supplementary file 1 [file nanomaterials-12-00100-s001.zip › nanomaterials-1496496 supplement.pdf]

nanomaterials

Supplementary materials

Solvent-induced assembly of one-patch silica nanoparticles into robust clusters, wormlike chains and bilayers

Bin Liu [1,2], Serge Ravaine [2] and Etienne Duguet [1*]

[1] Univ. Bordeaux, CNRS, Bordeaux INP, ICMCB, UMR 5026, 33600 Pessac, France; bin.liu-bin@u-bordeaux.fr

[2] Univ. Bordeaux, CNRS, CRPP, UMR 5031, 33600 Pessac, France; serge.ravaine@crpp.cnrs.fr

* Correspondence: etienne.duguet@icmcb.cnrs.fr; Tel.: +33-540-002-651

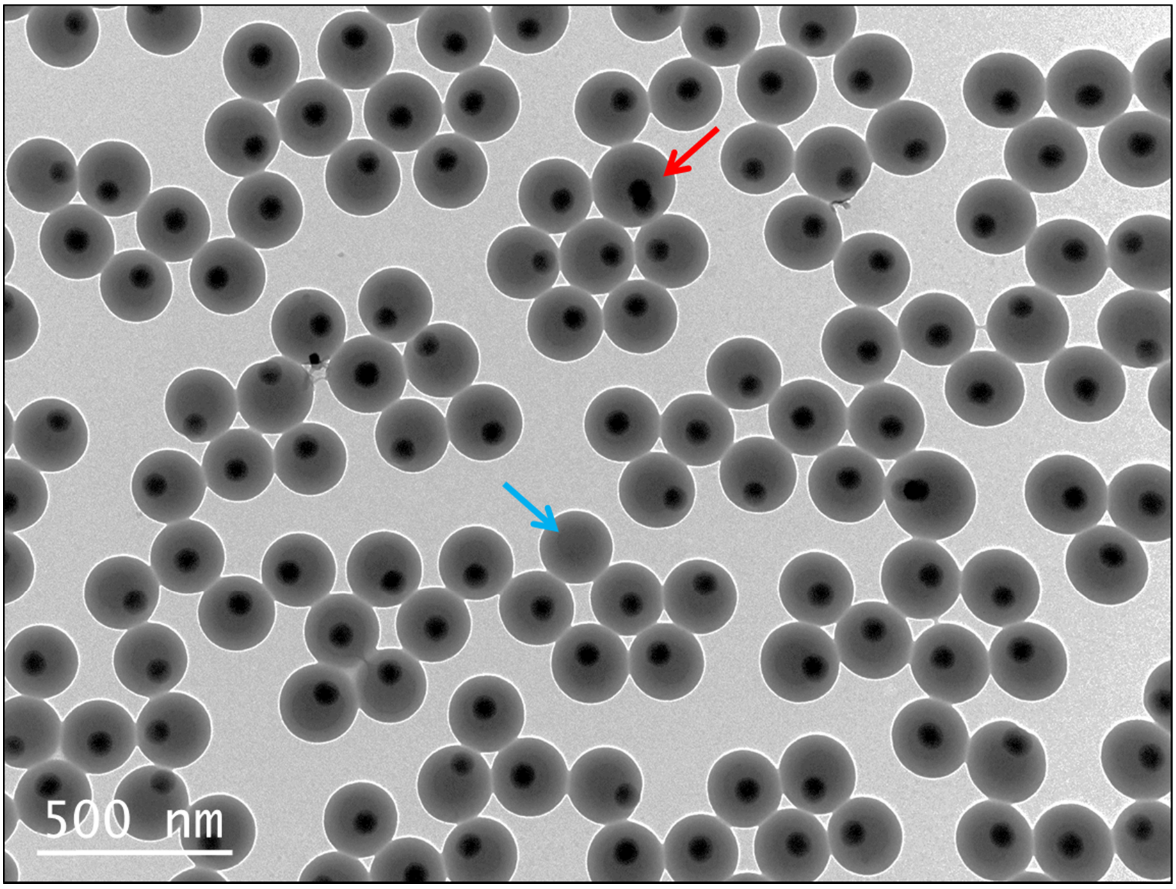

Figure S1. Representative TEM image of silica/PS monopod-like nanoparticles as obtained by emulsion polymerization of styrene in the presence of 60-nm silica seeds previously surface-modified with MMS at the nominal grafting surface density of 1 funct./nm^2. Both arrows evidence the presence of side-products: PS particle with two silica seeds (red arrow) and PS particle without any silica seed (blue arrow, without any consequence on the yield of the forthcoming synthesis of one-patch silica particles because of the PS dissolution stage). The measured morphology yield is 98 % with regard to the silica seeds.

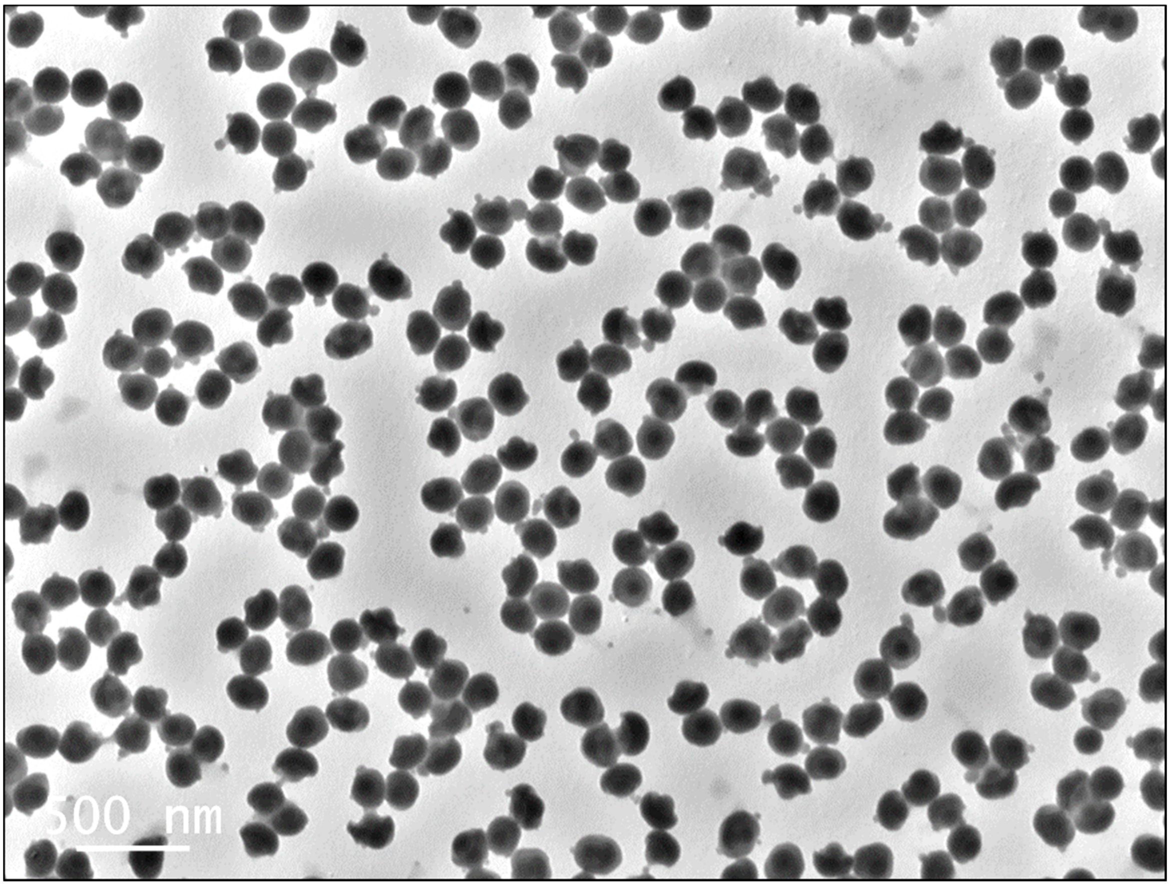

Figure S2. Representative low magnification TEM image of 145-nm silica particles with a single 100-nm PS patch (S = 0.69). Smaller particles are residual PS nanoparticles resulting from incomplete washing after dissolution in THF.

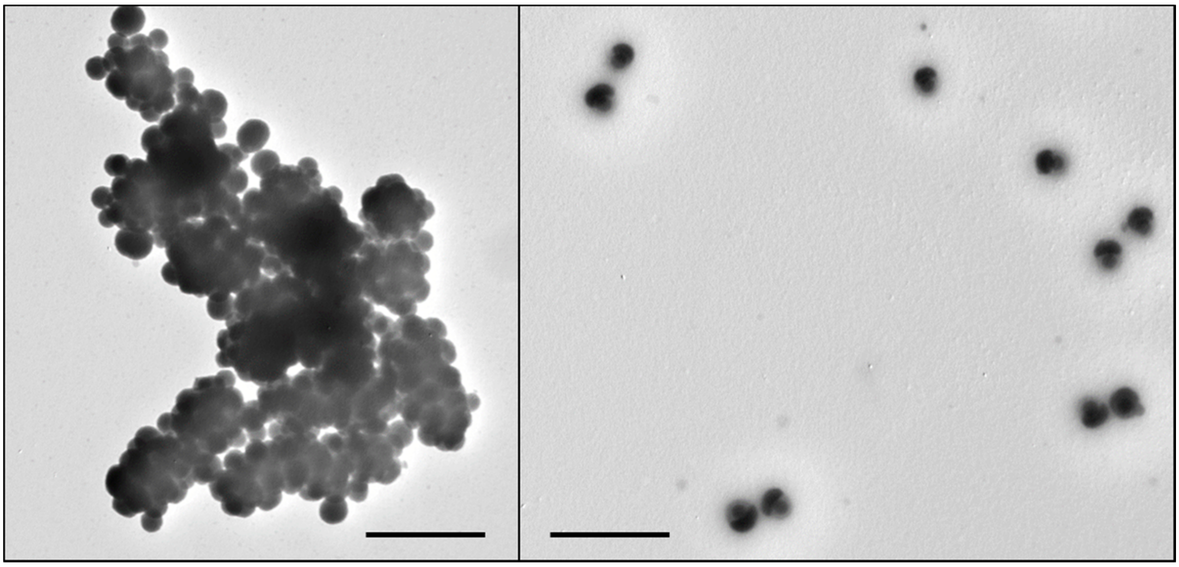

Figure S3. Representative TEM images of micelles obtained from 115-nm silica particles (S = 0.87) as obtained in a few days in equivolume THF/ethanol mixture (left) before and (right) 10 min after the addition of an additional quantity of THF to lower the ethanol fraction to 20 vol%. Scale bars: 500 nm.

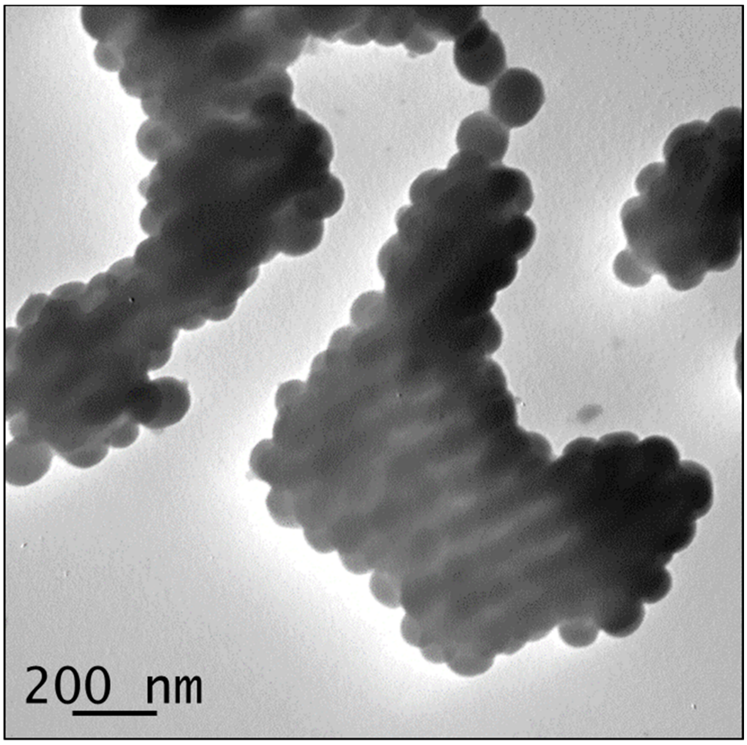

Figure S4. Representative TEM image of bilayers obtained from 85-nm silica particles (S = 1.18) after 90 min of incubation in equivolume THF/ethanol mixture under ultrasounds (Ultrasonic cleaning bath Fisherbrand™ FB15051 used at room temperature).

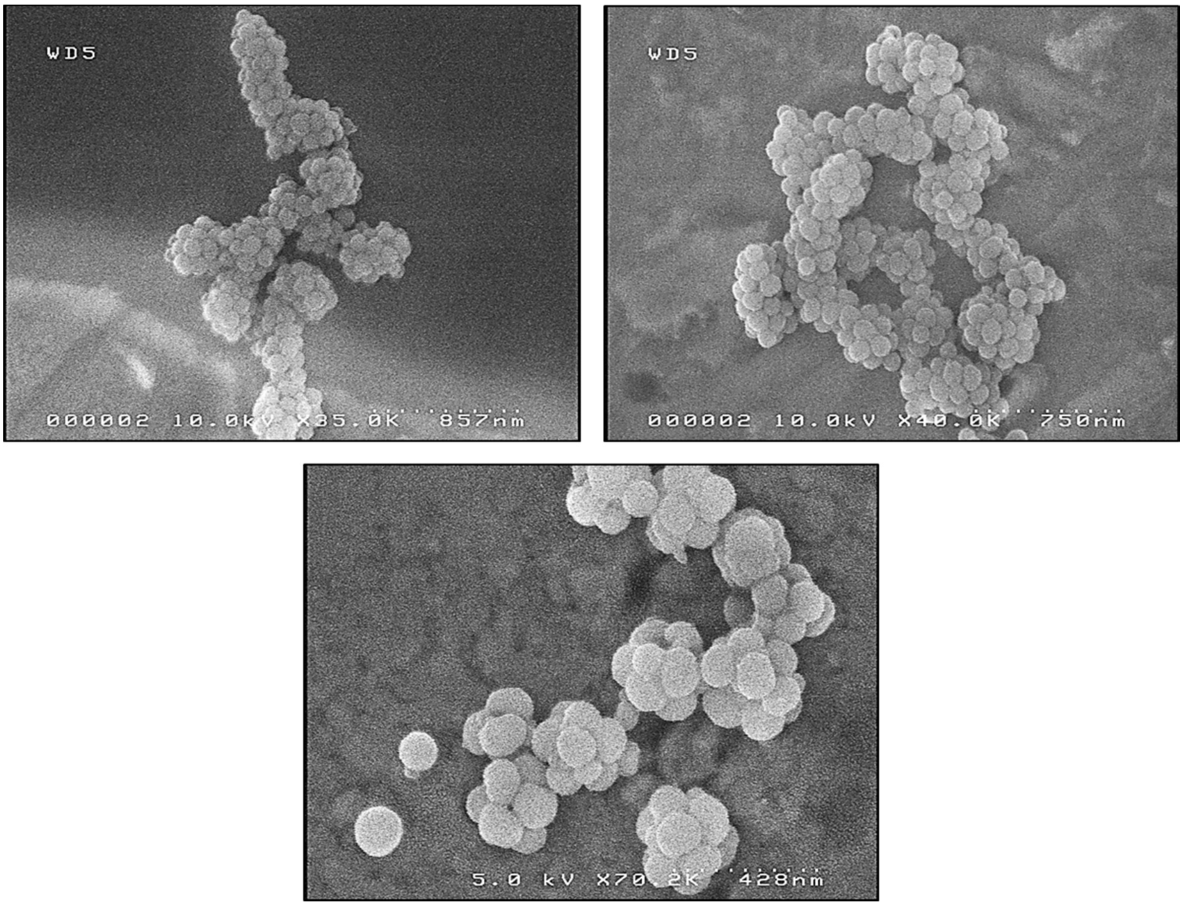

Figure S5. Representative scanning electron microscopy (SEM) images of the silica particles present in the incubation medium after 5 days. **Top:** spherical micelles and wormlike chains obtained from silica monomers with S = 0.87. **Bottom:** large clusters and spherical micelles obtained from silica monomers with S = 0.69. SEM images were obtained on Hitachi S-4500 microscope operating at 10 kV. Before analysis, samples deposited on a 300-mesh carbon coated copper grid, were coated with nm-thick layer of gold and palladium (10 %) with a SC7620 mini sputter coater.

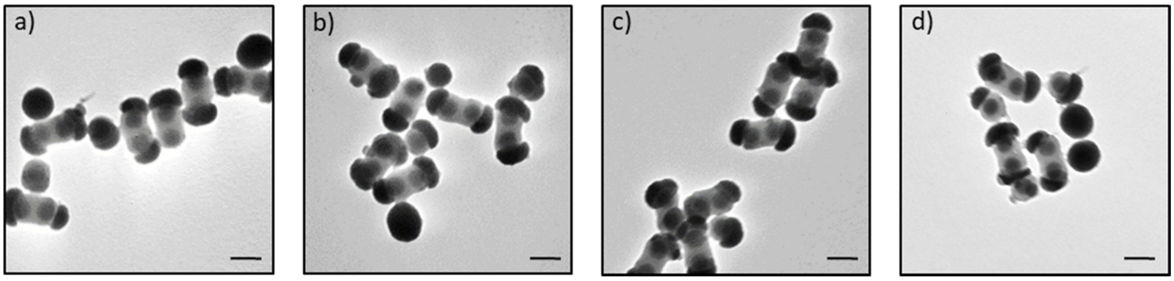

Figure S6. Representative TEM images of the silica dimers obtained from 85-nm silica particles (S = 1.18) after 3 h of incubation in equivolume THF/ethanol mixture at room temperature on a roller mixer, and after being diluted 20 times in ethanol and stored at 4°C for **a)** 20 h, **b)** 7 days, **c)** 32 days, and **d)** 52 days. Scale bars: 100 nm. This experiment shows that excess ethanol freezes the structure of the assemblies and preserves them over long periods.
